# Periodic Precipitation in Hele-Shaw Cells: Mechanism Insights from Experiments and Numerical Simulation Considering Layer Thickness Effects

**DOI:** 10.1021/acs.langmuir.5c03631

**Published:** 2025-11-13

**Authors:** Nobuhiko J. Suematsu, Yuhei Onishi, Masaki Itatani, Daishin Ueyama, István Lagzi

**Affiliations:** a Graduate School of Advanced Mathematical Sciences, Meiji University, 4-21-1, Nakano, Tokyo 164-8525, Japan; b Meiji Institute for Advanced Study of Mathematical Sciences (MIMS), Meiji University, 4-21-1, Nakano, Tokyo 164-8525, Japan; c Department of Chemistry, Faculty of Science, 12810 Hokkaido University, Sapporo 060-0810, Japan; d Faculty of Engineering, Musashino University, 3-3-3 Ariake, Koto-ku, Tokyo 135-8181, Japan; e Department of Physics, Institute of Physics, Budapest University of Technology and Economics, Műegyetem rkp. 3, Budapest H-1111, Hungary; f HUN-REN−BME Condensed Matter 592 Physics Research Group, Budapest University of Technology and Economics, Műegyetem rkp. 3, Budapest H-1111, Hungary

## Abstract

Liesegang patterns are self-organized macroscopic structures formed by precipitates. Traditional methods for producing these patterns rely on gel phases, which inhibit flow but introduce complications, such as undesired chemical interactions. This study presents a strategic approach utilizing a “Hele-Shaw cell”, consisting of two glass plates that create a narrow gap (30–225 μm). This setup prevents flow due to friction between glass surfaces without the use of a gel. We identified two critical thresholds for the gap thickness: one that inhibits flow and another that facilitates discrete precipitate bands. For aqueous solutions of CuCl_2_ and K_2_CrO_4_, these thresholds were determined to be 110 and 150 μm, respectively. Below the first threshold, the period of precipitate bands increased with a greater gap thickness. This thickness dependency was successfully reproduced using our proposed mathematical model based on the microscopic dynamics of precipitation processes instead of the phenomenological step function model commonly used. Our numerical simulations indicate that the nucleation rate of precipitates significantly influences the formation of bands, with the nucleation rate having an inverse effect on band spacing. It is assumed that the nucleation rate is proportional to the specific surface area of the glass plate, and thus, the practical nucleation rate was proportional to the inverse of the thickness. This research provides a simplified experimental method and a robust mathematical model for Liesegang pattern formation, potentially paving the way for the further exploration of self-organization applications.

## Graphical abstract

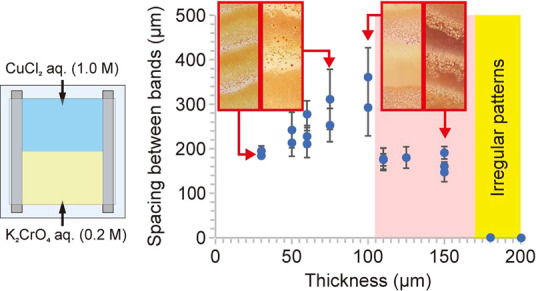

## Introduction

Self-organization and self-assembly are the structure and pattern formation phenomena that create complexity in animate and inanimate systems.^1^ These processes occur spontaneously without human intervention in thermodynamically open and closed systems. Reaction-diffusion systems are one emblematic and enormously studied example of self-organization in chemical systems.^2–4^ In this case, static or spatiotemporal patterns form because of the interplay between the diffusion and chemical reaction of the involved chemical species. Periodic precipitation (PP or the Liesegang phenomenon) is one of the subclasses of reaction-diffusion systems in which the initially spatially separated reagents generate precipitation zones in solid gels due to their diffusion and chemical reactions.^5–8^ The pattern can typically be formed in quasi-1D (test tube), 2D (thin gel layer), or 3D (spherical tube) systems depending on the geometry of the setup.^9^ In all cases, the precipitation structures are determined by the spatiotemporal characteristics of the chemical front generated by the diffusion flux of one reagent diffusing in the gel. In the usual setup, one reagent (inner electrolyte) is homogeneously distributed in the gel matrix, and the other (outer electrolyte) is placed in contact with the gel. The concentration of the outer electrolyte is greater (one or two orders) than the inner one, generating a traveling chemical front in the gel matrix.^7^

Several empirical regularities can characterize the formed precipitation structures. These are the spacing,^10^ Matalon–Packter,^11–13^ time, and width laws.^14^ Among them, the most universal and valid in many chemical systems producing PPs is the spacing law:

$$\underset{n\to \infty }{\lim}\;\frac{x_{n+1}}{x_{n}}=1+p$$ 1

where *n* is the band number, *x*_*n*_ and *x*_*n*+1_ are the distances of the *n* ^th^ and (*n* + 1)^th^ bands measured from the interface between media containing the outer and inner electrolytes, respectively, and *p* is the spacing coefficient.

The gel plays a critical role in pattern formation because these porous matrices have relatively large pore sizes, ranging between a few tens and a few hundred nm (depending on the chemical composition and concentration of the monomers).^15^ In this environment, the reagents and formed nuclei can diffuse. However, the bigger aggregates and precipitate particles cannot be produced because they are trapped in the gel matrix. This makes the pattern spatially static in a gravitational field. Namely, the created precipitation zones remained where they were formed. In other words, the gel prevents the sedimentation and formation of hydrodynamic instabilities. Therefore, designing and engineering PP in the pure liquid phase provides a significant challenge. Since the discovery of PP, all works and studies have exclusively used solid gels as reaction media.

Investigation of the formation of PPs is essential not only from the fundamental point of view but also from the applied science point of view. A series of recent studies showed that a reaction-diffusion setup can be used to synthesize not only exclusive inorganic precipitation particles but also other crystalline materials such as gold nanoparticles^16^ and metal–organic frameworks.^17^ The power of this approach originates from the sophisticated control of nucleation and growth regimes, driven by diffusion in the solid gel matrix. In addition, controlling the reaction media and experimental conditions can generate hierarchical and complex precipitation structures.^18–22^

Our previous paper presented that PP can be generated in a thin liquid layer confined in the Hele-Shaw (HS) cell.^23^ The spatiotemporal properties of the PPs we observed align with established empirical laws governing pattern formation, including the spacing, Matalon–Packter, time, and width laws. Furthermore, PPs were observed not only in the CuCrO_4_ system but also in other precipitation systems such as Ag_2_Cr_2_O_7_ and ZIF-8. We also conducted an experimental investigation to determine the critical gap distance (*d*) of HS cells required to form PPs. We discovered that the critical *d* aligned perfectly with the simple prediction based on the Rayleigh–Darcy number (*Ra*) and geometrical parameter (ε) of HS cells. The pattern formation occurred if ε^2^ *Ra* was smaller than unity (the system was in the Rayleigh–Darcy regime). However, when ε^2^ *Ra* was greater than unity, the fluid flow might have appeared, preventing the formation of periodic precipitation structures. However, the gap distance was realized at only 70, 120, 240, and 680 μm, and the band pattern was observed with only 70 and 120 μm in the gap distance. Therefore, the detailed experiments to uncover the qualitative thickness dependency are still under investigation.

Several models were developed in the past to describe the spatiotemporal evolution of the PPs. The models can be categorized into two categories: pre- and postnucleation models.^24–27^ In the prenucleation model, nucleation occurs only in the region where the product of the ion concentrations exceeds the solubility product of the precipitate (i.e., the nucleation is not continuous behind the traveling chemical front). In contrast, in the postnucleation model, the nucleation is spatially continuous and the reaction front leaves a constant concentration of nuclei. The precipitation occurs if the nuclei concentration reaches a threshold concentration. In both models, the mathematical description contains a step (Heaviside) function to produce distinct zones in space.^7^ It should be noted that the mathematical description does not include a step function in models with the nonreaction kinetic description of the precipitation (spinodal decomposition or competitive particle growth).^28–30^ The precipitation process occurs due to thermodynamic instability.

In this article, we systematically investigate the effect of the thickness of the liquid layer in an HS cell on the frequency of the precipitation structures and discuss the effect of the cover surface of the HS cell on the heterogeneous nucleation. We developed a new kinetic description of precipitation to elaborate on the experimentally observed trend. This kinetic model does not contain a step function, and the precipitation process occurs through the bistability encoded in the model.

## Experimental Methods

### Preparation of the Handmade Hele-Shaw Cell

The HS cell was created by using a slide glass, a cover glass, and thin silicone films with thicknesses (*d*) of 30, 50, and 75 μm (see Figure 1). The silicone films were stacked to realize various thicknesses for the HS cell: 30, 50, 60 (30 × 2), 75, 100 (50 × 2), 110 (50 + 30 × 2), 125 (50 + 75), 150 (75 × 2), 180 (75 × 2 + 30), 200 (75 × 2 + 50), and 225 (75 × 3) μm. The gap distances were experimentally estimated for three typical samples: 30, 75, and 150 μm (Figure S1).

**Figure 1:**
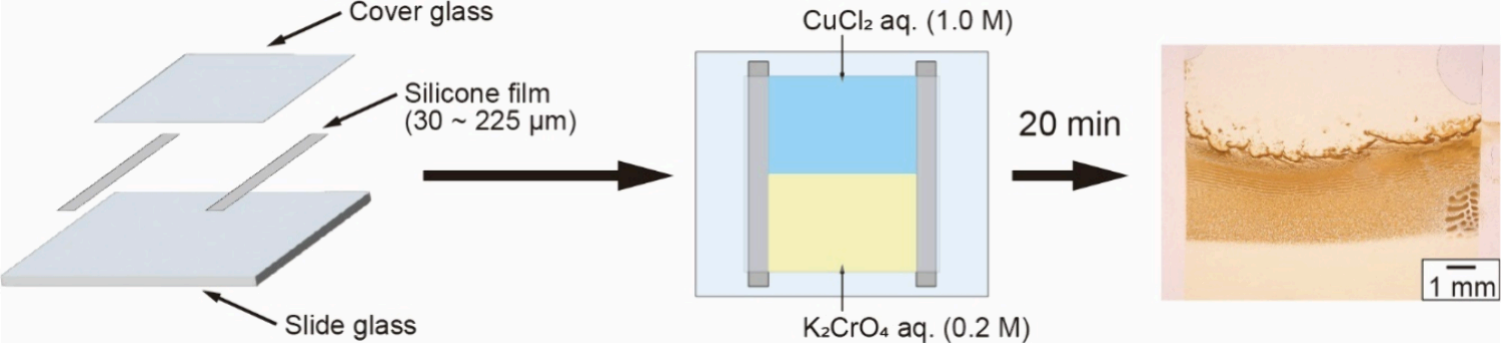
Schematic illustration of the Hele-Shaw (HS) cell preparation, whose thickness was controlled using silicone thin films. In the experiment, the silicone thin films of 30, 50, and 75 μm in thickness were used as spacers. These films were placed on a slide glass and covered with a cover glass. Next, two reactant solutions, 1.0 M CuCl_2_ and 0.2 M K_2_CrO_4_, were poured into the gap between the glasses from opposite ends. Within 20 min, a precipitation pattern began to form where the two solutions made contact.

### Pattern Formation and Analysis

The reactant solutions were a 1.0 M aqueous solution of copper­(II) chloride (CuCl_2_, Fujifilm Wako Pure Chem.) and a 0.2 M aqueous solution of potassium chromate (K_2_CrO_4_, Fujifilm Wako Pure Chem.). The solutions were poured into the narrow gap of the HS cell from opposite ends (Figure 1). To eliminate evaporation of the solutions, both edges were shielded using Vaseline. The chemical reaction occurred from the contact line of the two solutions, and precipitates were produced.

The precipitation patterns were observed using optical microscopy (VHX Keyence), which obtained snapshots and movies. The characteristic wavelength of the pattern was estimated by analyzing the snapshots, and the flow rate around the precipitation bands was measured from the movies. Both analyses were carried out using image analysis software ImageJ (NIH, USA).

## Results and Discussion

### Periodic Precipitate Bands Depending on the Thickness of the Liquid Phase

The aqueous solutions of CuCl_2_ and K_2_CrO_4_ were poured into the narrow gap between the slide glass and cover glass (borosilicate). A precipitation reaction occurred at the interface between the solutions, forming CuCrO_4_ precipitates. The entire process was observed using an optical microscope (see Movie S1). The observations showed that small particles or nuclei formed a short distance away from the previous precipitate band. Over time, the number of particles increased. The distribution of particle number density gradually spread toward the K_2_CrO_4_ solution. After a few minutes, the spreading process slowed and particle growth at the edge of the precipitate band became dominant. Eventually, the growth process also stopped and a new band began to form, indicating the start of the nucleation process a short distance away from the edge of the previous band.

In the observed phenomena within the HS cell, bands were identified with a thickness of up to 150 μm in the gap (Figure 2a). These bands exhibited more irregular patterns when the gap exceeded 180 μm, as shown in Figure S2. The average distance between these bands was 190 μm at a gap thickness of 30 μm, and this spacing linearly increased with a gap thickness of up to 100 μm, at which point the spacing reached 340 μm. The data were analyzed using linear regression, resulting in the fitting curve shown in Figure 2b. The slope was 1.84 ± 0.27, the intercept was 130 ± 9 μm, and the correlation coefficient was 0.916. Additionally, we prepared the box plot showing more statistical information (Figure S3). A notable decrease in spacing to 176 μm was observed when the gap thickness was 110 μm. Beyond this point, the spacing remained nearly constant, even as the gap thickness increased to 150 μm, as illustrated in Figure 2b. It should be noted that to characterize the pattern structure, we calculated the average distance between the bands instead of calculating the spacing coefficient (eq 1). The distance was measured from the peak of the brightness (Figure 2a-ii). On the other hand, it was hard to estimate the width of bands because the zones were not distinct. Furthermore, the irregular banding pattern was observed when the gap exceeded 180 μm. In this case, the bands were still visible, but the spacing was irregular, and the band shapes were not straight. These patterns may result from complex flow caused by sedimentation. Our results reveal that there are two threshold thicknesses: (I) 110 μm and (II) 180 μm (Figure 2b). The latter threshold well agrees with that reported in the previous paper, where the band pattern was observed up to 120 μm but disappeared over 240 μm.^23^

**Figure 2:**
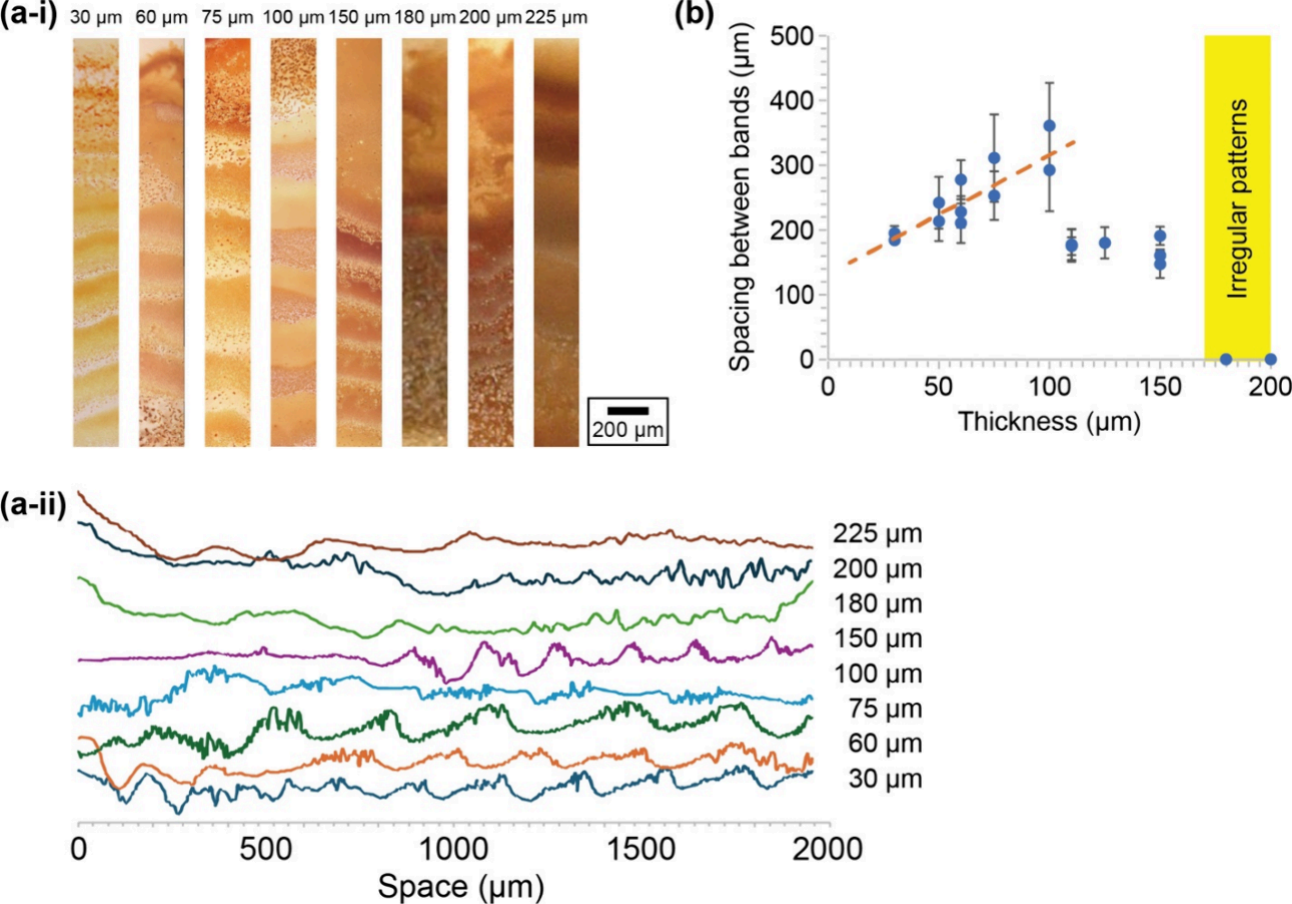
(a) Periodic precipitate bands of CuCrO_4_ depending on the thickness of the liquid phase. (i) Photographs and (ii) brightness plotted against space are shown. The thickness ranged from 30 to 225 μm, with variations in combination with silicone films. (b) Spacing distance between the precipitate bands depending on the thickness. The error bars represent the standard deviation for each sample, with 3–5 data points included. The orange broken line indicates the fitting curve originating from the linear regression. The experiments were carried out two to three times for each thickness, and the results were plotted separately. Thick conditions higher than 180 μm induced irregular patterns and made it hard to measure the spacing.

### Flow around the Precipitate Band

In this study, a liquid–liquid system was used as a reactor designed to inhibit macroscopic flow through the frictional forces exerted by the top and bottom glasses. Therefore, the effect of friction decreases with an increase in the thickness of the HS cell, and macroscopic flow could be generated beyond a critical thickness threshold. To clarify the effect of the flow on the precipitate band pattern, the flow around the precipitate band was observed (Figure 3a). Here, the small precipitate particles of CuCrO_4_ served as indicator particles. The supplementary movies (Movie S2–Movie S5) indicate the flow near the precipitate band. In addition, deviation among 10 s image sequences also shows the flow (Figure 3b). The rapid movement of these small particles was documented through the trajectories presented in the deviation images. The findings explicitly showed that no flow was observed with the HS cell gap narrower than 100 μm (Figure 3b-i,ii). However, a noticeable onset of flow was detected in the HS cell with a gap exceeding 110 μm (Figure 3b-iii,iv). The focus of the images was set at the top of the solution. Thus, our observations suggest that originally, there is no flow; however, if particles that can sediment are present, then they can create flow only in larger-gap HS cells (as illustrated in Figure 3c-ii).

**Figure 3:**
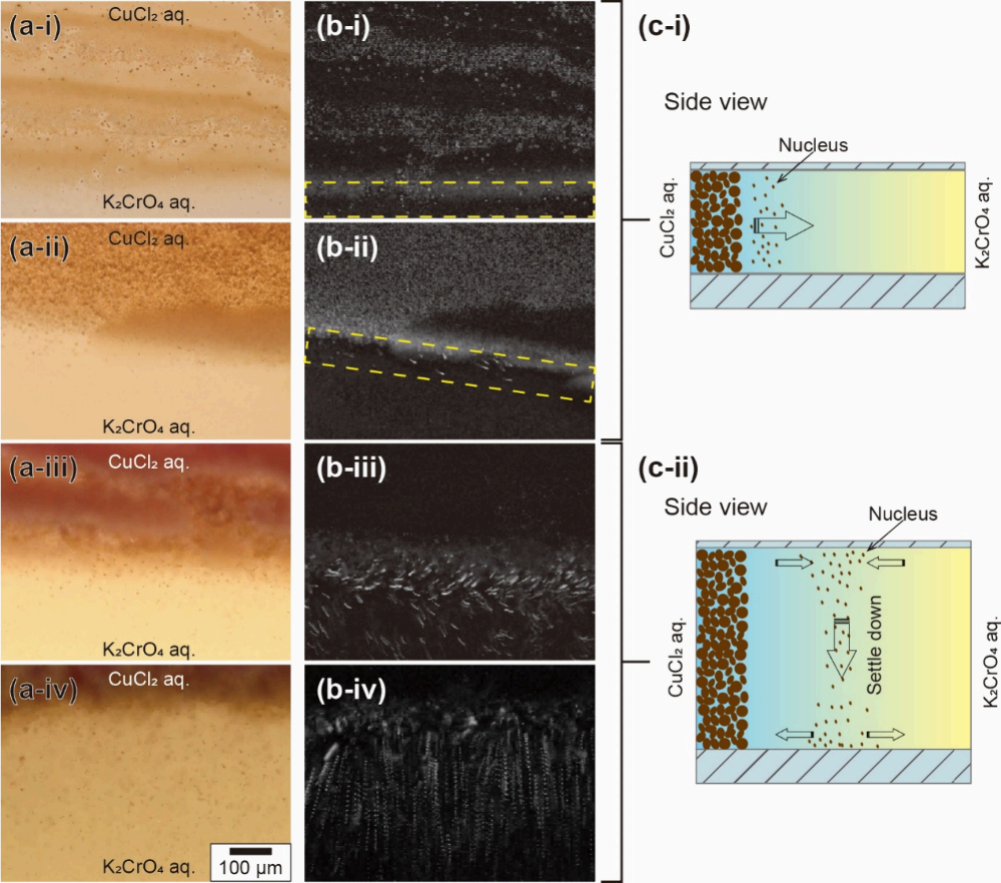
Analysis of the fluid dynamics around the precipitate bands. (a) Snapshots of the precipitate bands. (b) Deviations during 10 s, as analyzed through ImageJ. The thicknesses of the liquid layers were (i) 30, (ii) 75, (iii) 150, and (iv) 225 μm. (c) Schematic illustrations of the fluid dynamics around the precipitate band under (i) thin and (ii) thick depth conditions.

### Mathematical Model

We developed a mathematical model based on a simple chemical reaction to explain the mechanism behind the discrete formation of precipitate bands. There have been numerous mathematical reports on Liesegang patterns, most of which use a step function to reproduce precipitation processes. This involves the production of precipitates when concentrations exceed a certain threshold, known as the “solubility product”. Recently, we introduced a straightforward mathematical model for precipitation processes that does not rely on a step function.^31^ Our model considers an autocatalytic process and utilizes a cubic function for the precipitation process, effectively reproducing the observed solubility product in the experiments. Careful experimental observations have confirmed the autocatalytic process in precipitation.^32–34^ In this study, we apply this concept and consider the following simple chemical reactions and precipitation processes:

$$\{\begin{array}{c} U+V\to W\left(\mathrm{aq}\right)\\ W\left(\mathrm{aq}\right)⇌C\\ C\to S \end{array}\begin{array}{c} \left(R1\right)\\ \left(R2\right)\\ \left(R3\right) \end{array}$$

where U and V are reactants, W is the product that is dissolved in the aqueous phase as molecules even though it is difficult to dissolve, C is the nucleus and colloid particles of the product, and S is the solid particles (precipitate). The chemical species are denoted with capitals U, V, W, C, and S. It is assumed that the chemical reaction (R1), dissolution of the nucleus and colloids (inverse of R2), and precipitation (R3) progress occur through mass action, while the growth process of nuclei and colloids (R2) progresses through an autocatalytic process. The growth process should be considered using the Smoluchowski equations.^35^ However, solving the complex Smoluchowski equations is quite challenging. Therefore, in the initial stage of this complex aggregation process, we approximate the growth of the nucleus and colloids using an autocatalytic process. Specifically, we consider only three processes for the production and growth of the nucleus: (i) nucleation, (ii) adhesion of molecules to the nucleus and colloids, and (iii) collision and adhesion of the nucleus and colloids (Figure 4a). Here, we assume that the rates of processes (i) and (ii) are considered as mass action, while the rate of process (iii) is represented by a nonlinear process originating from, for example, the collision of colloid particles, which is assumed to be proportional to *wc* ^2^.

**Figure 4:**
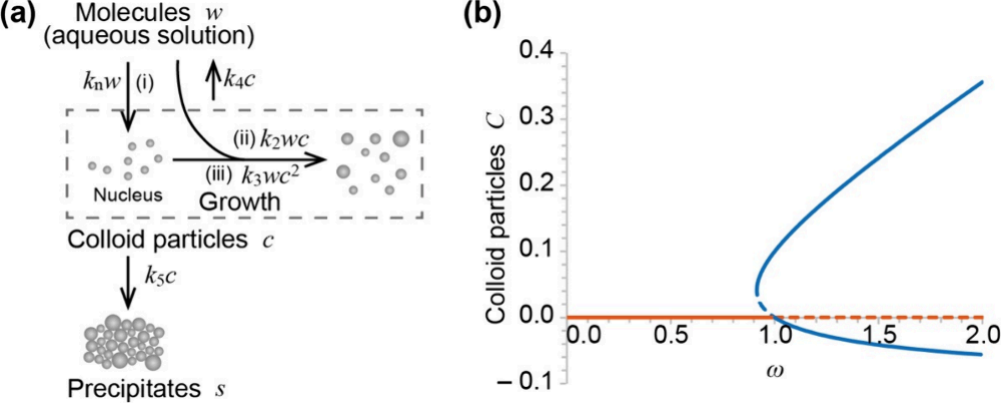
(a) Schematic illustration of the mathematical model for the precipitation reaction. (b) Bifurcation diagram showing the equilibrium points associated with colloid particles C under the condition κ > τ. The bifurcation parameter is the total concentration of the precipitate materials (ω), namely, the sum of the concentration of the aqueous solution W and colloids C. In the diagram, solid and broken lines indicate stable and unstable branches, respectively.

Then, we can construct the following partial differential equations.

$$\{\begin{array}{c} \frac{\partial u}{\partial t}=D\frac{\partial^{2}u}{\partial x^{2}}-k_{1}uv\\ \frac{\partial v}{\partial t}=D\frac{\partial^{2}v}{\partial x^{2}}-k_{1}uv\\ \begin{array}{c} \frac{\partial w}{\partial t}=D\frac{\partial^{2}w}{\partial x^{2}}+k_{1}uv-k_{2}wc-k_{3}wc^{2}-k_{n}w+k_{4}c\\ \frac{\partial c}{\partial t}=D_{c}\frac{\partial^{2}c}{\partial x^{2}}+k_{2}wc+k_{3}wc^{2}+k_{n}w-k_{4}c-k_{5}c\\ \frac{\partial s}{\partial t}=k_{5}c \end{array} \end{array}$$ 2

where *u*, *v*, *w*, *c*, and *s* are the concentrations of U, V, W, C, and S, respectively, and *k*_1_, *k*_2_, *k*_3_, *k*_4_, *k*_5_, and *k*_n_ are the positive constants concerned with the reaction rate. The diffusion coefficients of molecules U, V, and W are the same (*D*), and that of the colloid particles C is a lower value (*D*_c_). The definitions for each parameter are summarized in Table 1. The precipitates are assumed to have no diffusion. From the above equations, the following dimensionless equations are obtained.

$$\{\begin{array}{c} \frac{\partial U}{\partial T}=\frac{\partial^{2}U}{\partial X^{2}}-UV\\ \frac{\partial V}{\partial T}=\frac{\partial^{2}V}{\partial X^{2}}-UV\\ \begin{array}{c} \frac{\partial W}{\partial T}=\frac{\partial^{2}W}{\partial X^{2}}+UV-\left(1+\kappa C\right)CW-\eta W+C\\ \tau \frac{\partial C}{\partial T}=\frac{\varepsilon }{\tau }\frac{\partial^{2}C}{\partial X^{2}}+\left(1+\kappa C\right)WC+\eta W-C-\gamma C\\ \frac{\partial S}{\partial T}=\gamma C \end{array} \end{array}$$ 3

where the parameters are set as follows:

$$\begin{array}{c} t=t_{0}T,\;u=u_{0}U,\;v=v_{0}V,\;w=w_{0}W,\;c=c_{0}C,\;s=s_{0}S,\;x=x_{0}X,\;\\ t_{0}=\frac{k_{2}}{k_{1}k_{4}},\;u_{0}=v_{0}=w_{0}=\frac{k_{4}}{k_{2}},\;c_{0}=\frac{k_{1}k_{4}}{k_{2}^{2}},\;x_{0}=\sqrt{\frac{k_{2}D}{k_{1}k_{4}}},\;s_{0}=\frac{k_{2}}{k_{1}},\;\\ \eta =\frac{k_{2}k_{n}}{k_{1}k_{4}},\;\gamma =\frac{k_{5}}{k_{4}},\;\tau =\frac{k_{1}}{k_{2}},\;\varepsilon =\frac{D_{c}}{D},\;\mathrm{and}\;\kappa =\frac{k_{1}k_{3}k_{4}}{k_{3}^{2}} \end{array}$$

**Table 1:** Relationships between Parameters and Processes

notation	corresponding process
*k*_1_	the rate constant of reaction (process R1)
*k*_2_	the growth rate constant of colloids originating from the adsorption of molecules to colloids (the linear term of the process R2)
*k*_3_	the growth rate constant of the autocatalytic process for the colloid formation (the nonlinear term of the process R2)
*k*_4_	the dissolution rate constant of a colloid particle (the reverse process of R2)
*k*_5_	the rate constant of precipitation (the process R3)
*k*_n_	the nucleation rate constant (the part of the process R2)
*D*	diffusion coefficient of the molecules, U, V, and W
*D*_c_	diffusion coefficient of the colloids

Dimensionless parameters are set in italic capitals. Here, *k*_1_, *k*_2_, and *k*_4_ represent the rate constants for the reaction, growth, and dissolution, respectively. These values are assumed to be greater than the nucleation rate, *k*_n_. Therefore, the value of η is supposed to be negligible.

Stability analysis can reveal the characteristics of nonlinear precipitation processes. To understand the properties of the equation, we consider a closed system that includes only *W* and *C* (see the Supporting Information). Therefore, the chemical reaction between U and V and the precipitation process producing S are neglected. The initial conditions for *W* and *C* are *W*_ini_ and *C*_ini,_ and their sum ω is constant. The time evolutions of *W* and *C* result in conservative systems. Therefore, the time evolution of *W* and *C* can be described by the following one-parameter ordinary differential equation. The following equation is obtained using dimensionless equations (eq 3). The derivation is explained in the Supporting Information.

$$\tau \frac{dC}{dT}=(1+\kappa C)(\omega -\tau C)C-C$$ 4

where ω is the total mass of W and C, and the nucleation process, η*W*, is neglected by assuming that its value η is negligibly small. The equilibrium point *C*_0_^*^ = 0 exists at all time. Additionally, the value of *C*_±_^*^ satisfying (1 + κC*)­(ω – τC*) – 1 = 0 is also the equilibrium point that exists under the condition with ω is larger than the threshold value, $2\sqrt{\tau /\kappa }-\tau /\kappa$ . The parameters τ and κ describe the characteristic rate of precipitate production and the production rate of a nonlinear process relative to a linear one, respectively. The bifurcation diagram strongly depends on the relationship between κ and τ, and a bistable region exists with κ > τ (Figure S4). Here, for simplicity, we consider the condition with κ > τ, where there is a bistable region, hereafter. This condition exhibited discrete precipitate patterns easily. However, it is noted that the condition with κ < τ also showed a band pattern with κ > 1.0 (Figure S5). Thus, a bistable region is not necessary for band formation. The bifurcation diagram indicates that *C*_0_^*^ is the unique equilibrium point and is stable with a low value of ω; in this case, no colloid particles are in a stable condition (Figure 4b). With the increase in ω and over the threshold value, the saddle-node bifurcation occurs and *C*_±_^*^ appears, in which *C*_+_^*^ and *C*_–_^*^ are stable and unstable equilibrium points, respectively. In this condition, *C*_0_^*^ is still a stable equilibrium point and no colloids are generated unless a big perturbation is applied. With a further increase in ω, the stable *C*_0_^*^ and unstable *C*_–_^*^ cross at ω = 1, and transcritical bifurcation occurs. Therefore, *C*_0_^*^ becomes unstable, and the state transitions to *C*_+_^*^. It means that colloid particles are generated under the condition of ω > 1. These behaviors resemble a step function, usually used to reproduce precipitation phenomena.

The precipitation processes are numerically calculated in one-dimensional space using the above-mentioned equations. The boundary conditions are the no-flux except for *U*(0, *t*), where the Dirichlet boundary condition with the value of *U*_0_ is applied. As the initial condition, we set *V* = *V*_0_ for all of the fields homogeneously and others set as 0. A typical example is shown in Figure 5a (Movie S6). In the beginning, the reactant U diffuses from the left-hand side, where the concentration of U is kept at *U*_0_ as the boundary condition, and W is gradually produced due to the chemical reaction with V. As a result, the concentration of U decreases monotonically and approaches 0 as it moves from the boundary. In the region with a nonzero value of *U*, the value of *V* becomes almost 0, and the *W* is produced. Thus, the chemical reaction occurs only at the narrow region with nonzero values of both *U* and *V*; we refer to this region to as the “reaction front”. Due to the reactions and diffusion, the reaction front propagates to the right-hand side. The nucleus C is produced by W, and a part of C goes back to W and the others grow and gather, resulting in precipitates S. As it shows in the bifurcation diagram (Figure 4b), the production rate of C exceeds the back-reaction to W with the total mass of C and W, which is ω, overcoming the threshold value 1, and the precipitate band is produced. Due to the diffusion of W, a depletion region appears around the band, and the next band is produced at a discrete position (see Figure 5a and Figure S6). These processes agreed with the well-known mechanism of Liesegang pattern formation.^7^

**Figure 5:**
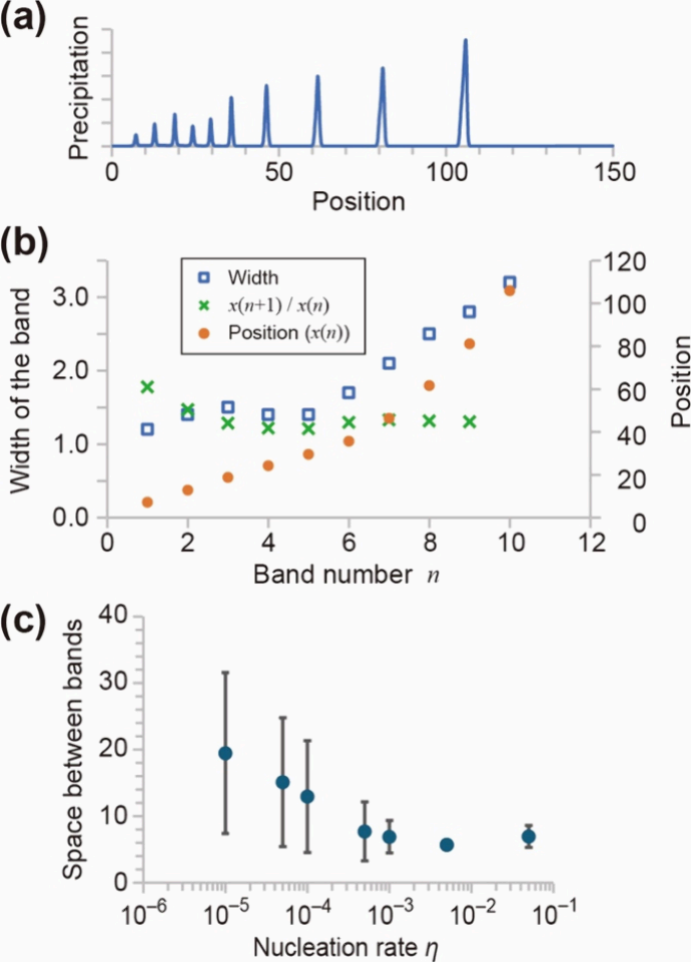
Numerical results. (a) Typical profile of the precipitate. (b) Characteristics of the precipitate bands. The parameters are set to *u*_0_ = 10, *v*_0_ = 3, η = 0.005, γ = 0.01, τ = 5.0, ε = 0.1, and κ = 10. The length of the reaction domain *L* is 200, the time step d*t* is 0.005, the spatial step d*x* is 0.1, and the time length of the calculation is 5000. The temporal difference was made by the Euler method, and the spatial difference was made by the central difference. (c) Spacing between the initial five bands depending on the nucleation rate (η). The average value of five spacings was plotted against the nucleation rate with standard deviations as error bars.

After enough time, a series of precipitation bands were generated. A typical result is shown in Figure 5a, where the discrete formation of bands of precipitates is observed. The width of each band linearly increases with the band number *n* beyond the fifth band, known as the width law (Figure 5b).^14^ Here, the band is defined as those with an amount of the precipitate, *S* value, greater than 1. The widths of the band are constant if *n* < 4, and this deviates from the width law. Additionally, the positions of bands are plotted against the band number *n*. The ratio of distances of two consecutive bands measured from the liquid–liquid interface (junction point of the electrolytes) (*x*_*n*+ 1_/*x_n_*) is a constant value if *n* > 3, which is known as the spacing law (eq 1 and Figure 5b). The bands close to the junction point of the electrolytes cannot fulfill the width and spacing laws. This observation in the numerical simulations is in good accordance with the results observed in experimental systems.^36^ Furthermore, our numerical calculations agree with the Matalon–Packter law, which suggests that the *p* value of eq 1 depends on the initial values of *U*_0_ and *V*_0_ (Figure S7). Those results indicate that our suggested model well reproduces the typical properties of the Liesegang pattern.

In our experimental system, the spacing was almost constant, independent of bandwidth, and the bandwidth was hard to estimate due to intrinsic band edges. The former is well reproduced with numerical simulation during the initial five bands (Figure 5b). However, it is difficult to compare or discuss the width because there is no estimation in the experimental results. Additionally, the slide and cover glasses might support and enhance the nucleation. In other words, the nucleation rate (η) depends on the specific surface area of the glasses against the volume of reactant solution, and thus, as the gap distance increases, the volume of the reaction solution also increases, while the surface area remains constant. This leads to a decrease in the nucleation rate with an increase in the thickness. Here, the effect of η on the period of bands is investigated numerically. Similar to the analysis of experimental results, the spacing of the initial five bands is measured with a change in the value of η. Figure 5c indicates that the period decreases with an increase in η. Namely, a high nucleation rate induces a low period of the bands. This tendency agrees well with our experimental observations, which is the thickness dependency of the spacing with a narrow gap (Figure 3b). With the narrow gap up to 110 μm, the spacing increased with the thickness (Figure 3b). Decreasing the thickness corresponds to increasing the surface ratio of the glass, which will enhance nucleation, namely, the high value of η. Therefore, the thickness dependency observed in our experiments can be explained by the decrease in the effective nucleation rate.

In this study, the parameters *k*_1_–*k*_5_ have not yet been experimentally estimated, making it challenging to verify our model quantitatively. However, assuming that our model and parameter set match the experimental conditions, the reactants’ diffusion coefficient is estimated at 1.25 × 10^–9^ m^2^ s^–1^ (see the SI for the details of the estimation process). It is noted that this estimation does not validate the overall model correctness. To confirm the model and parameters, physicochemical parameters and comparisons between simulations and experiments are necessary. This will be the focus of future work. Additionally, our model cannot currently specify the threshold thickness based on our experimental findings. To better understand these results, the model should be extended to incorporate fluidic behaviors in the vertical direction, requiring a two-dimensional model. In this study, we focus only on the HS cell, where no flow occurs. Future work will involve developing a 2D model that includes vertical sedimentation.

### Mechanism

In contrast to well-established gel systems, the liquid–liquid systems inhibit flow primarily due to the frictional interactions between the glass surfaces. As a result, when the system’s thickness exceeds a critical limit, it fails to maintain flow resistance, leading to the disruption of patterns (Figure 2 and Figure S2). Our observations indicate that the sedimentation of the precipitate particles is the origin of the flow. Namely, density instability is dominant in this system. In this manner, there are the following three stages depending on the thickness of the gap:

I. No sedimentation occurs due to the narrow gap between the glass plates. At this stage, there is no flow, not only in vertical but also in horizontal directions, and thus, the condition is quite similar to that of a typical gel system. The experimental results are presented in Figure 2a (up to 100 μm) and Figure 3i,ii.
II. Slow sedimentation occurs during band formation, and the simple flows are induced as shown in Figure 3iii. This stage was observed with a gap between 110 and 150 μm.
III. Sedimentation of the particles induces a fast flow. The flow perturbs the concentration gradients, and thus, irregular patterns were generated (Figure 3iv and Figure S2).

The linear relationship between the gap and spacing observed in stage I has been previously reported in gel systems.^37^ Previous experiments used a stamping system with a thin gel similar to ours. However, the circumstances were quite different. In that system, the thickness affected the outer solution since the outer solution was supplied from the stamp to the reaction field, namely, the thin gel, in proportion to the stamp’s surface area. This meant that the supply rate/diffusion flux was inversely proportional to thickness, assuming a constant stamp surface area. Our numerical simulation, shown in the Supporting Information, indicates that the spacing increases as a function of the inverse of the outer concentration (Figure S7b). This supports the idea that the linear relationship between thickness and spacing in the previous work was due to the outer solution’s concentration. In other words, this is the manifestation of the Matalon–Packter law. In our case, however, the outer solution concentration was kept constant, generating a constant diffusion flux of the outer electrolyte. While our results resemble those of previous studies, the underlying mechanism is different.

Our mathematical model precisely replicates the distributed band pattern seen in experiments under no-flow conditions. Movie S1 displays the banding behavior, showing both distinct particles and brownish shading. We interpret the brownish shading as crystal particles located in the middle or bottom regions of the HS cell. The focus of Movie S1 was on the top of the glass surface, so particles in the middle and bottom layers appeared out of focus and showed shading; we confirmed this by adjusting the focus. Additionally, crystals persisted in the depletion zone, which is the region between the bands. We believe that the concentration of CuCrO_4_ is low in the depletion zone, which prevents nucleation. However, if small nuclei form, then they can grow. As a result, a few larger crystals are observed in the depletion zone. Our numerical calculations also suggest that a small amount of precipitate forms in the depletion zone, indicated by a nonzero value of *p* observed between the bands (Movie S6).

The flow observations (Figure 3a-i,ii) indicate that no flow was generated when the gap was narrower than 100 μm. Under these conditions, the flux remained nearly constant. However, the spacing of the bands increased with the gap in the HS cell (Figure 2b). This relationship can be attributed to the properties of the glass surfaces. The glass surfaces differ from the bulk solution and may facilitate nucleation. As a result, narrow-gap HS cells exhibited a higher nucleation rate due to the increased specific surface area of the glass in contact with the solution phase. Numerical simulations show that a higher nucleation rate leads to a lower band spacing (Figure 5c). Thus, the increased spacing observed with larger gaps can be understood because of a lower effective nucleation rate.

As discussed above, the nucleation mainly occurs on the glass surfaces, and thus, precipitates produced on the top glass settle down with the thickness of space overcoming the threshold value. Our experimental findings indicate a threshold value of around 110 μm (Figure 2b). For gaps greater than 110 μm, flow away from the precipitate band was observed in the upper region of the solution (Figure 3b-iii). The flow might work by enhancing the flux of the reactants. According to the Matalon–Packter law, increased flux results in reduced spacing between bands. The data illustrated in Figure 2b qualitatively support this phenomenon. Thus, the observed decrease in band spacing at larger gaps can be attributed to the enhanced flux generated by the sedimentation of small precipitate particles (Figure 3b-iii). In this region, a thicker gap induces a higher flux and a lower nucleation rate. The former decreases the band spacing, whereas the latter increases it. We considered that these two opposite effects generate a constant value of band spacing from 110 to 150 μm. Further increasing the gap may lead to more complex flow dynamics, resulting in the disruption of periodic banding in thick HS cells exceeding 180 μm (Figure 2 and Figure S2). Our proposed mechanism is based on the hypothesis that the surface enhances nucleation processes. To verify this, we needed to compare the banding behavior across different surface configurations. Preliminary experiments using a hydrophobic glass surface showed that aqueous solutions did not penetrate the narrow gap of the HS cell. While assessing the surface condition dependency is crucial, it requires more careful modifications to the experimental setup. This will be addressed in future work. Additionally, the concentrations and chemical components also change the reaction rate, nucleation rate, and density of the colloids. Therefore, the thickness dependency of the spacing and the two threshold thicknesses may also depend on the chemical components, which are topics for future work.

## Conclusions

In conclusion, this study demonstrates that the thickness of the HS cells significantly influences the formation of self-organizing Liesegang bands. We observed that thicker HS cells, compared to those measuring 110 μm, generated flow around the bands due to the sedimentation of precipitate particles. This flow not only shortened the spacing between bands but also produced more intricate patterns. These results highlight both limitations of HS reaction cells in obstructing flow. Additionally, under the narrow conditions, our experiments reveal that an increased gap between HS cells leads to a wider spacing of the precipitate bands, primarily due to a reduced nucleation rate resulting from the surface ratio of the glasses against bulk solutions. The standard deviation of the spacing in our results is relatively larger than that in the previous work, indicating that our system is affected by stochastic behavior due to processes such as random nucleation and sedimentation. These points are disadvantages of our system from the viewpoint of engineering applications. Conversely, our system does not need to account for the effects of gel or gelation conditions, which is an advantage. The consistency of our findings with numerical calculations using a novel mathematical model devoid of step functions underscores the robustness of our approach. The results indicate the potential of our model in elucidating Liesegang patterns without relying on step functions. Overall, we believe that the insights gained from the HS reaction system and our mathematical model can significantly advance the understanding of self-organization phenomena associated with various nonlinear chemical reactions.

## Supplementary Material















## Data Availability

The data that support the findings of this study are available from the corresponding authors upon reasonable request.

## Notes

Additional experimental results and details of mathematical approaches (PDF)
Movie S1: in situ observation of generating precipitate bands of CuCrO_4_ (MP4)
Movie S2: real-time movie for the flow around the precipitate band (gap distance: 30 μm) (MP4)
Movie S3: real-time movie for the flow around the precipitate band (gap distance: 75 μm) (MP4)
Movie S4: real-time movie for the flow around the precipitate band (gap distance: 150 μm) (MP4)
Movie S5: real-time movie for the flow around the precipitate band (gap distance: 225 μm) (MP4)
Movie S6: time evolutions for the concentrations of each element obtained by numerical calculation (MP4)
