# Supplementary material for: Periodic Precipitation in Hele-Shaw Cells: Mechanism Insights from Experiments and Numerical Simulation Considering Layer Thickness Effects
Source: Langmuir. 2025 Nov 13;41(46):31105–13. doi: 10.1021/acs.langmuir.5c03631 (PMC12704908; doi:10.1021/acs.langmuir.5c03631)
Supplement: Supplementary file 1 [file la5c03631_si_001.pdf]

Supplementary Information

Periodic Precipitation in Hele–Shaw Cells: Mechanism Insights from Experiments and Numerical Simulation Considering Layer Thickness Effects

Nobuhiko J. Suematsu,*[a, b] Yuhei Onishi,[a] Masaki Itatani,[c] Daishin Ueyama,[d] and István Lagzi*[e, f]

[a] Graduate School of Advanced Mathematical Sciences, Meiji University, 4-21-1, Tokyo 164-8525, Nakano, Japan
[b] Meiji Institute for Advanced Study of Mathematical Sciences (MIMS), Meiji University, 4-21-1, Tokyo 164-8525, Nakano, Japan
[c] Department of Chemistry, Faculty of Science, Hokkaido University, Sapporo 060-0810, Japan
[d] Faculty of Engineering, Musashino University, 3-3-3 Ariake, Koto-ku, Tokyo 135-8181, Japan
[e] Department of Physics, Institute of Physics, Budapest University of Technology and Economics, Műegyetem rkp. 3, H-1111 Budapest, Hungary
[f] HU-REN–BME Condensed Matter Physics Research Group, H-1111, Műegyetem rkp. 3, Budapest, Hungary

1. Legends for movies

Movie 01. In situ observation of generating precipitate bands of $CuCrO_4$. The aqueous solutions of $CuSO_4$ and $KCrO_4$ were at the bottom and top of the movie, respectively. The video rate is 20 times faster than the real time.

Movie 02 – 05. The real-time movies for the behaviors around the precipitate band. The small particles of $CuCrO_4$ acted as indicator particles. The thicknesses of the HS cells were (Movie 02) 30 μm, (Movie 03) 75 μm, (Movie 04) 150 μm, and (Movie 05) 225 μm, respectively.

Movie 06. Time evolutions for the concentrations of each element were obtained by numerical calculation. The parameters are set to $U_0 = 10$, $V_0 = 3$, $\eta = 0.005$, $\gamma = 0.01$, $\tau = 5.0$, $\varepsilon = 0.1$, and $\kappa = 10$, which are the same as that of Figure 5a in the main text.

2. Estimation of the gap distance

The gap distance was controlled by using thin silicon films. We used three films with different thicknesses displayed: 30, 50, and 75 μm. To estimate the gap distance, three typical HS cells were prepared, and various volumes of pure water were penetrated the gap. The volume of water was controlled using a micropipette. Then, photos of the HS cells were taken, and the area occupied by water was estimated using ImageJ. From the volume and surface area, the gap distance was calculated. The estimated value of the gap distance was slightly smaller than the displayed one (Figure S1). The fitting curve indicates a slope of 0.96.

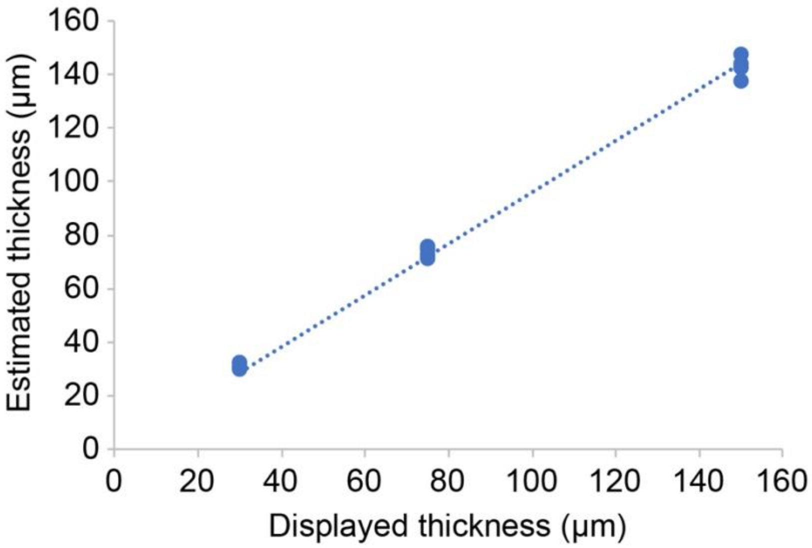

Figure S1. The estimated thickness of the HS cells against the displayed one.

3. Irregular patterns observed with thick Helle-Show (HS) cell

Clear precipitate bands with narrow gaps were observed in Helle-Show (HS) cells. Conversely, a complex band pattern emerged in thick HS cells with gap thicknesses exceeding 180 μm. In these thicker HS cells, discrete bands were also observed, but their

shapes were bent, and bands of varying periods and widths overlapped (Figure S2). These irregular patterns were characteristic of thick HS cells with gaps over 180 μm.

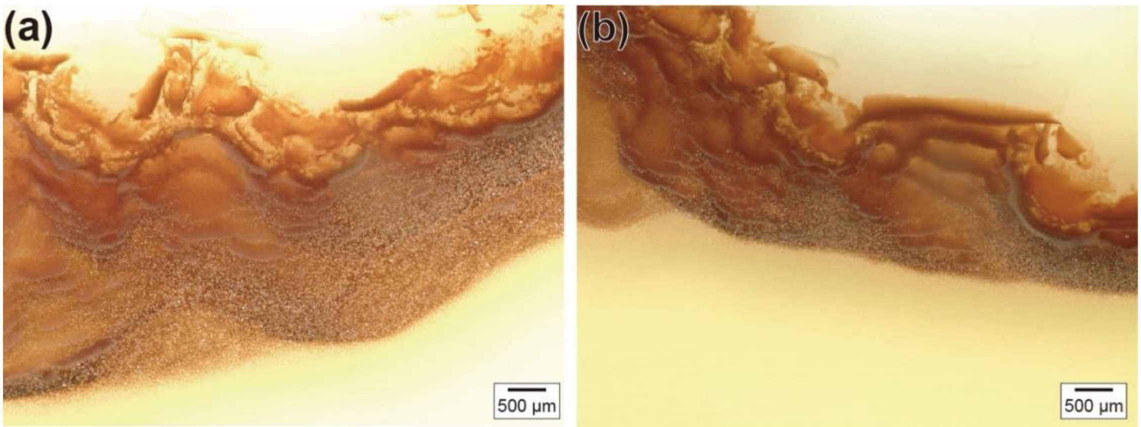

Figure S2. Irregular precipitate patterns produced in thick Helle-Show (HS) cells. The thickness was **(a)** 200 μm and **(b)** 225 μm.

4. Statistical analysis of the spacing between the bands

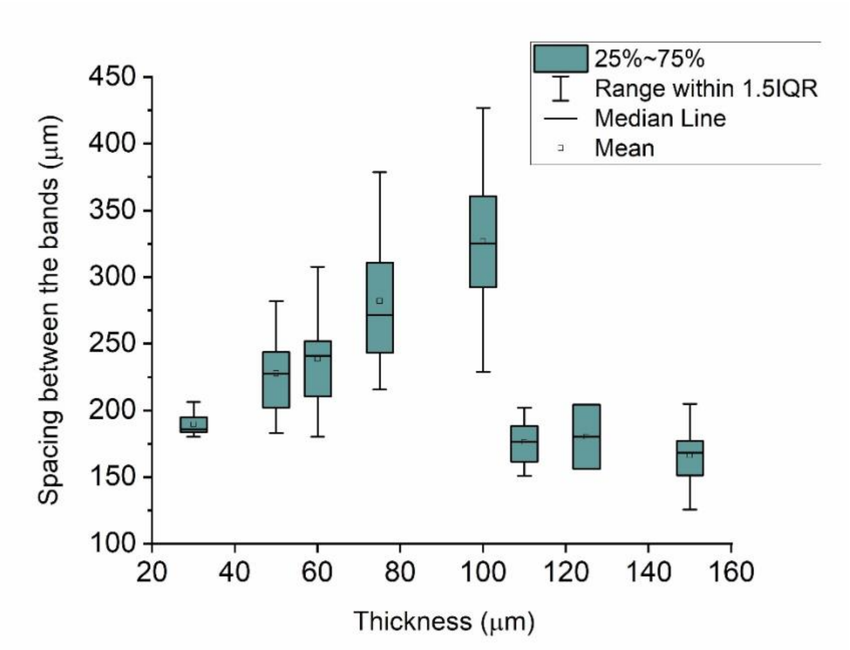

Figure S3. Box plot showing the relationship between the spacing between the bands and the thickness of the HS cell. The boxes indicate the interquartile range (IQR, 25–75%), whiskers represent values within 1.5×IQR, horizontal lines mark the median, and squares denote the mean values.

5. Stability analysis for precipitation processes

In this study, we suggested a mathematical model for precipitation processes that does not use the step function. In our model, instead of the step function, the autocatalysis process is considered for the growth process of the colloid particles (see Figure 4a in the main text). To consider the precipitation processes, we focus on the dynamics of only W and C, namely the following differential equations:

$$\begin{cases} \frac{dW}{dT} = -(1+\kappa C)CW - \eta W + C \\ \tau \frac{dC}{dT} = (1+\kappa C)CW + \eta W - C \end{cases} \tag{S1}$$

This is the conservative system: dW/dT + τdC/dT = 0. It means that $W + \tau C$ is constant, independent of time. Therefore, the constant $\omega = W + \tau C$ is introduced, and W in the differential equation of C is replaced with ω and C. Then, the following one-parameter ordinally differential equation is obtained.

$$\begin{gathered} \tau \frac{dC}{dT} = f(C), \\ f(C) = (1+\kappa C)(\omega - \tau C)C - C, \end{gathered} \tag{S2}$$

where the effect of the nucleation process (ηW) is neglected due to the value of η is small. The equilibrium points can be calculated to satisfy dC/dT = 0. The trivial solution is C_0^* = 0, which is always an equilibrium point. The other equilibrium points are the solution of $(1 + \kappa C)(\omega - \tau C) - 1 = 0$, which are

$$C_\pm^* = \frac{\kappa\omega - \tau \pm \sqrt{(\kappa\omega-\tau)^2 + 4\tau\kappa(\omega-1)}}{2\tau\kappa}. \tag{S3}$$

The equilibrium points $C_\pm^*$ exist under the condition with $(\kappa\omega+\tau)^2 > 4\tau\kappa$, and the saddle node bifurcation occurs at $\omega = 2\sqrt{\tau/\kappa} - \tau/\kappa$, where $C_\pm^* = 1/\sqrt{\kappa\tau} - 1/\kappa$. At this point, the bistable region appeared only with the condition $C_\pm^* > 0$, *i.e.*, $\kappa > \tau$ (Figure S4).

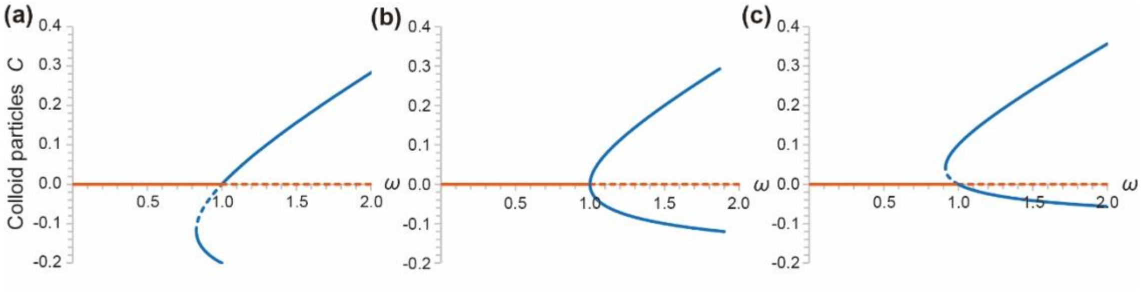

Figure S4. Bifurcation diagrams depending on κ. **(a)** $\kappa < \tau$, **(b)** $\kappa = \tau$, and **(c)** $\kappa > \tau$. The orange and blue lines indicate C_0^* and $C_\pm^*$, respectively. The solid and broken lines mean stable and unstable branches.

Based on the linear stability analysis, the stability of the equilibrium point can be determined by the sign of df/dC at $C = C^*$.

$$\frac{df}{dC} = (1 + \kappa C)(\omega - \tau C) + \kappa C(\omega - \tau C) - \tau C(1 + \kappa C) - 1 \tag{S4}$$

In the case of C_0^*, $C^* = 0$, (df/dC) = $\omega - 1$. Therefore, C_0^* is stable with $\omega < 1$. Next, in the case of $C_\pm^*$,

$$\left(\frac{df}{dC}\right)_{C_\pm^*} = \frac{1}{2\kappa\tau}\left[4\kappa\tau - (\kappa\omega + \tau)^2 \mp (\kappa\omega - \tau)\sqrt{(\kappa\omega + \tau)^2 - 4\kappa\tau}\right]. \tag{S5}$$

Here, we consider the condition with $\kappa > \tau$, where the bistable region exists. To exist the $C_\pm^*$, $(\kappa\omega + \tau)^2 - 4\kappa\tau \geq 0$ is required, and thus, the sum of the first and second terms of the right-hand side of eq. S5 is negative. Attritionary, the sign of $(\kappa\omega - \tau)$ is positive under the condition of $\kappa > \tau$. Therefore, (df/dC) is always negative for C_+^*, which is a stable equilibrium point. On the other hand, the sign of (df/dC) for C_-^* depends on ω. The necessary condition for stable C_-^* is that $(\kappa\omega + \tau)^2 - 4\kappa\tau > (\kappa\omega - \tau)\sqrt{(\kappa\omega + \tau)^2 - 4\kappa\tau}$, which means that $\omega > 1$.

Therefore, with the condition $\kappa > \tau$, the bifurcation diagram shown in Figure S4c is

obtained. This bifurcation diagram indicates that with low ω, C_0^* is a unique and stable equilibrium point. With ω over $2\sqrt{\tau/\kappa} - \tau/\kappa$, saddle-node bifurcation occurs and $C_\pm^*$ appears addition with C_0^*, where C_0^* and C_+^* are stable and C_-^* is an unstable equilibrium points. Further, an increase in the ω induces transcritical bifurcation at $\omega = 1$, where the stabilities of C_0^* and C_-^* exchange, thus, C_0^* becomes an unstable equilibrium point.

5. The effect of the autocatalysis process

The discrete bands are generated with precipitation reactions eq. 3 in the main text. As mentioned above, the precipitation processes are characterized by the relationship between κ and τ, which is the effect of a nonlinear process. Thus, the precipitation pattern also depends on the κ. The period of the discrete bands increases with κ, and finally, no band is produced with $\kappa = 1$ (Figure S5). Here, $\tau = 5.0$, thus, there is no bistable region with $\kappa < 5.0$, however, discrete bands are produced with $1.2 < \kappa < 5.0$ conditions. These results indicate that the discrete precipitation bands are generated without a bistable region; namely, step function-like dynamics are unnecessary for the discrete band pattern. However, the band pattern cannot be observed with a small value of κ. Therefore, an autocatalytic process of colloid formation is necessary to produce the Liesegang pattern.

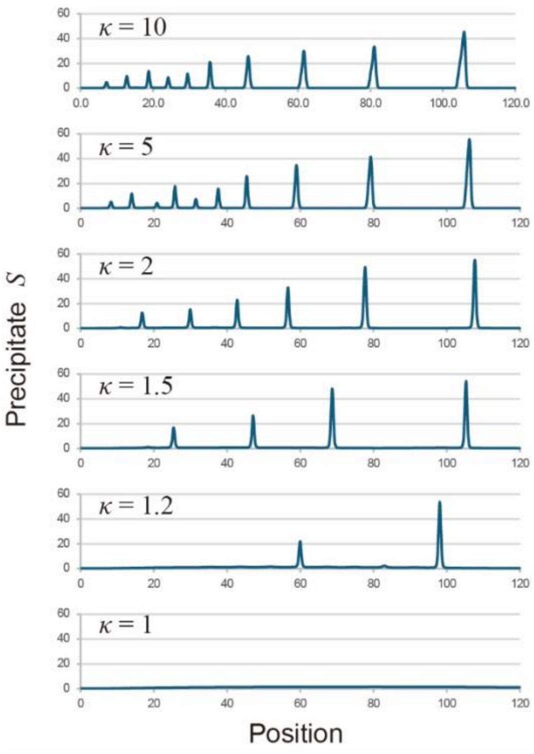

Figure S5. Precipitate patterns depending on κ. The parameters of numerical calculations are $U_0 = 10$, $V_0 = 3$, $\eta = 0.005$, $\gamma = 0.01$, $\tau = 5.0$, and $\varepsilon = 0.1$. The length of the reaction field L is 200, the time step dt is 0.005, the spatial step dx is 0.1, and the time length of the calculation is 5000.

6. Mechanism for the formation of discrete bands

The numerical results show the time evolution of the concentration profiles for W, C, and ω (Figure S6). The time evolution indicates the mechanism for generating the discrete bands. As is shown in Figure S3c, the equilibrium point C_0^* = 0 becomes unstable with the ω over 1, and C is produced. The concentration profile ω shown in Figure S6c indicates that ω drastically and locally increases at the position where ω over 1. At this position, W changes to C, and thus, W decreases and C increases locally (Figures S6a and S6b). The rapid reaction generates large concentration gradients of W and C. Thus, W diffuses to the position where the rapid reaction occurs. At the same time, C also diffuses

from the same position, but the diffusion coefficient of C is low, and thus the diffusion effect is negligible. As a result, the value of ω increases locally instead ω of decreases in other regions (Figure S6c). Namely, the discrete band of C originates from the difference in W and C diffusion coefficients.

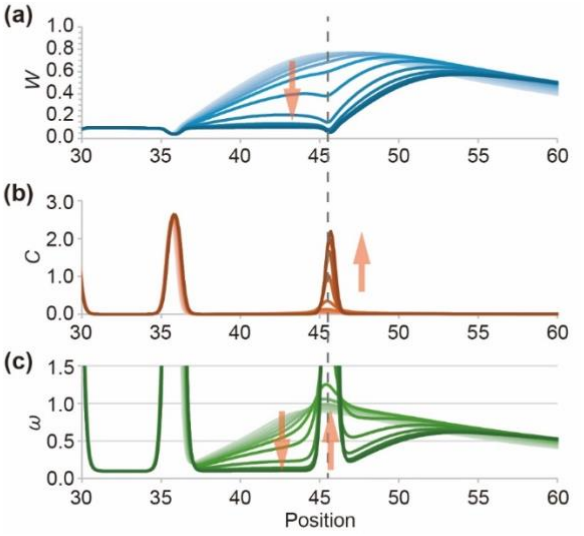

Figure S6. Time evolution of concentration profiles, **(a)** W, **(b)** C, **(c)** ω (= $W + \tau C$). The orange arrows indicate the changing direction of concentrations in time. The time progresses from light- to dark-colored lines.

7. Concentration dependency

The concentration of the inner (V_0) and the outer (U_0) solutions strongly affects the pattern of precipitation bands. The following spacing law usually estimates the patterns;

$$\frac{x(n+1)}{x(n)} = 1 + p. \tag{S6}$$

Our model can also be checked by the p value. With the increase in both V_0 and U_0, p values decrease (Figure S7). It is well known that the concentration dependency of the p

value obeys the Matalon-Packter (MP) law.

$$p = G(V_0) + F(V_0)\frac{1}{U_0}. \tag{S7}$$

Our numerical simulation shows that our model also complies with the MP law. The p values decrease as U_0 increases (Figure S7a). As shown in Figure S7b, p is inversely proportional to U_0, which is consistent with the MP law (Eq. S7). Furthermore, both the slope and the intercept, representing $F(V_0)$ and $G(V_0)$, decrease as V_0 increases. These results indicate that our proposed model effectively replicates the Liesegang pattern.

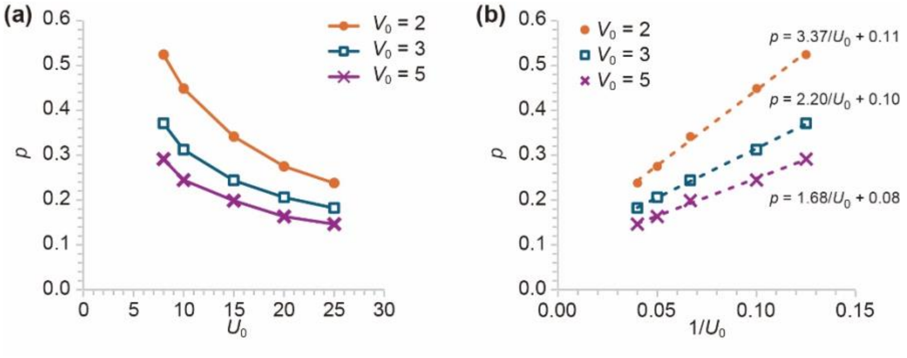

Figure S7. The p values depending on the values of U_0 and V_0.

8. Comparing the experimental and numerical results

Assuming our model and parameter set match the experimental conditions, the diffusion coefficient of the reactants is estimated to be 1.25×10^{-9} m² s^{-1}. Experimental data showed that the distance between bands was roughly 300 μm (Figure 2 in the main text) and the interval between band formation was about 200 s (Movie 01). Conversely, in simulations, these values were 6 and 100, respectively, resulting in estimates for x_0 and t_0 of approximately 50 μm and 2 s. Since the diffusion coefficient D is defined as

x_0^2/t_0, the value of diffusion is 1.25×10^{-9} m² s^{-1}, which appears reasonable for typical molecular diffusion. It is important to note that this estimate relies on the assumption that our parameter set and model are accurately verified against the experimental setup.
